# Supplementary material for: Pharmacokinetic, Metabolomic, and Stability Assessment of Ganoderic Acid H Based Triterpenoid Enriched Fraction of Ganoderma lucidum P. Karst
Source: Metabolites. 2022 Jan 21;12(2):97. doi: 10.3390/metabo12020097 (PMC8876931; doi:10.3390/metabo12020097)
Supplement: Supplementary file 1 [file metabolites-12-00097-s001.zip › metabolites-1423742-supplementary.pdf]

Supplementary Data

Figure S1: Representative HPLC chromatogram ganoderic acid H

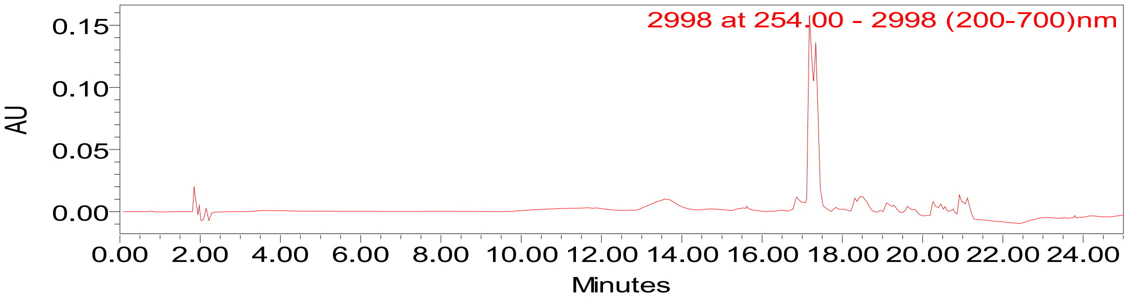

Figure S2: Representative HPLC chromatogram ganoderic acid H in mixture of other standards

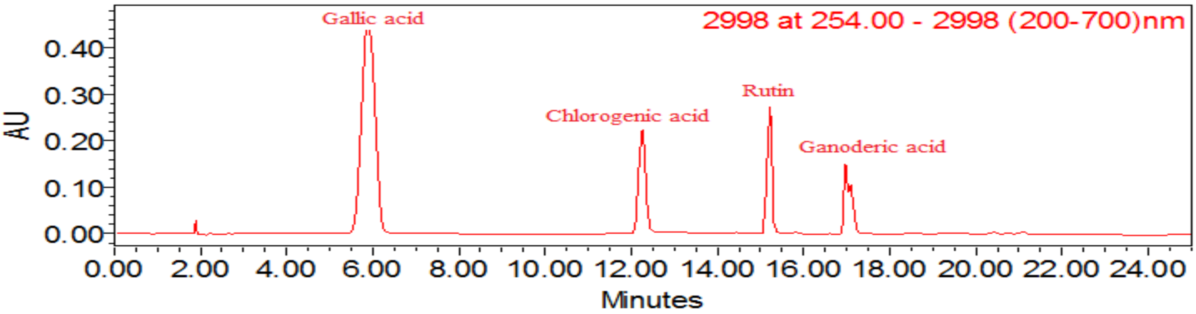

Figure S3: Representative HPLC chromatogram of TEF

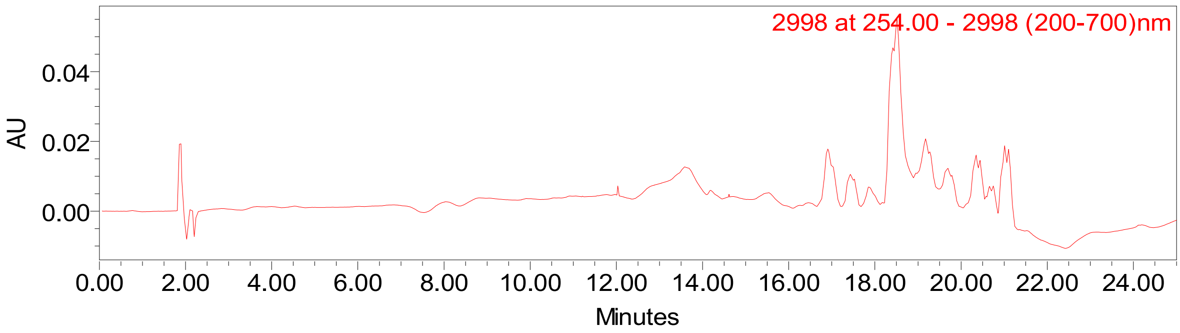

Figure S4: UV spectrum of ganoderic acid H

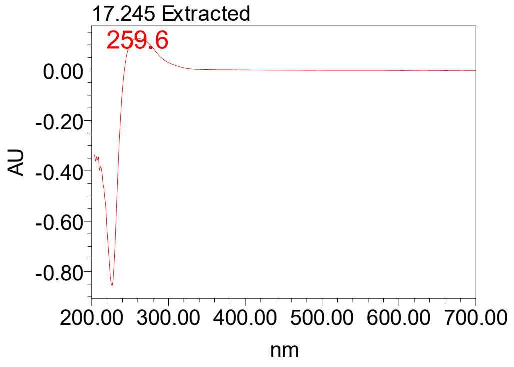

Figure S5: Representative UHPLC chromatogram of TEF

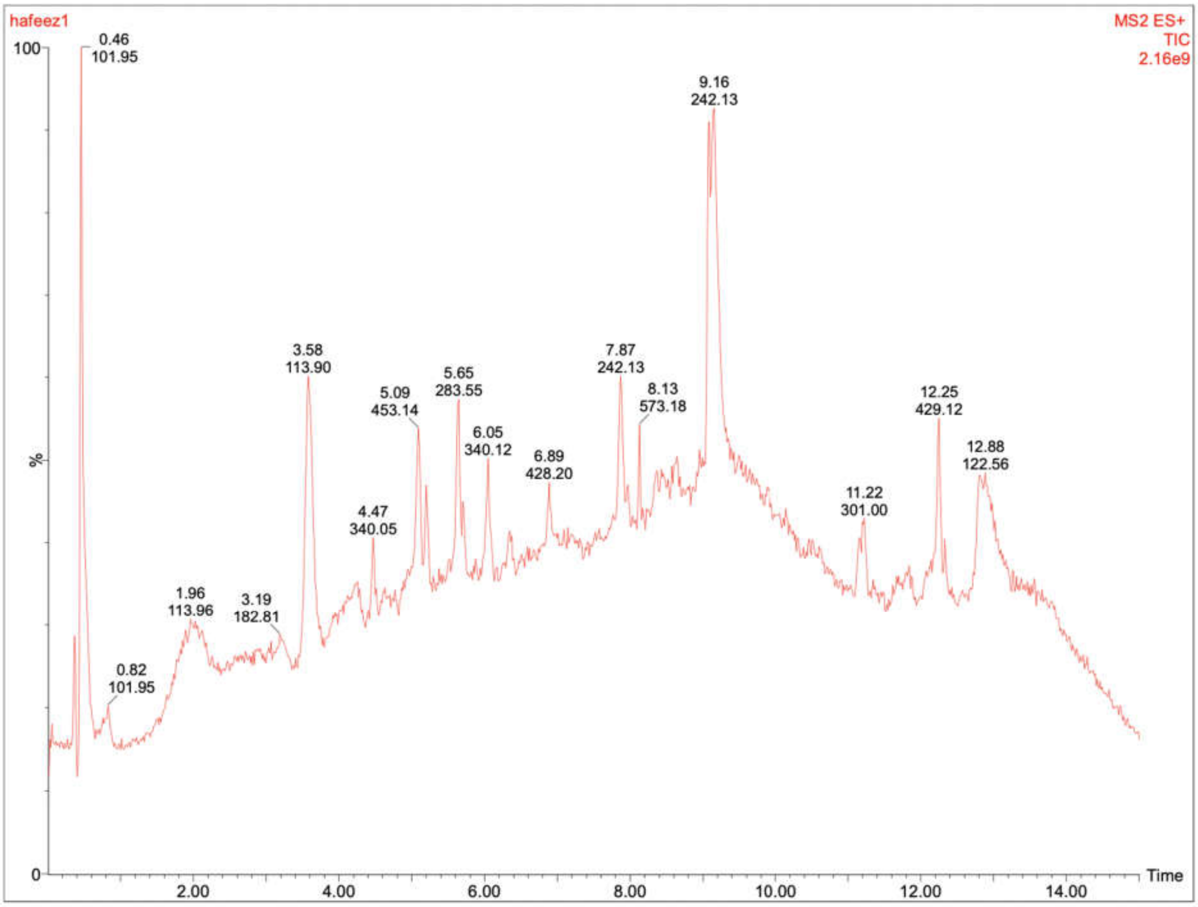

Figure S6: UPLC-MS chromatogram of TEF extracted at MS 573 showing of GA-H at R_t 8.14 min.

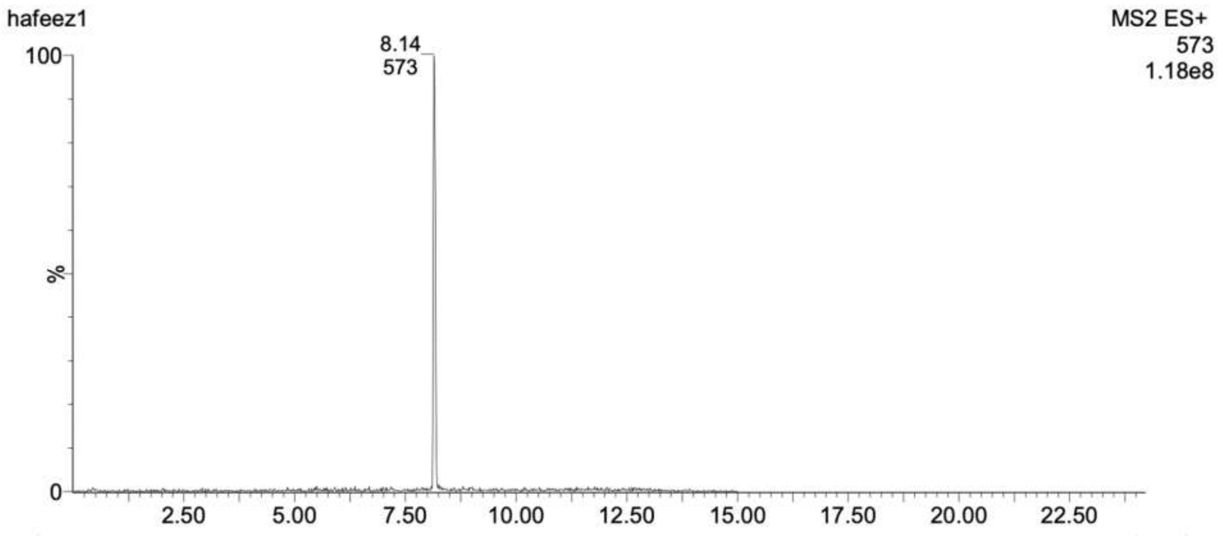

Figure S7: Representative GC-MS chromatogram of TEF.

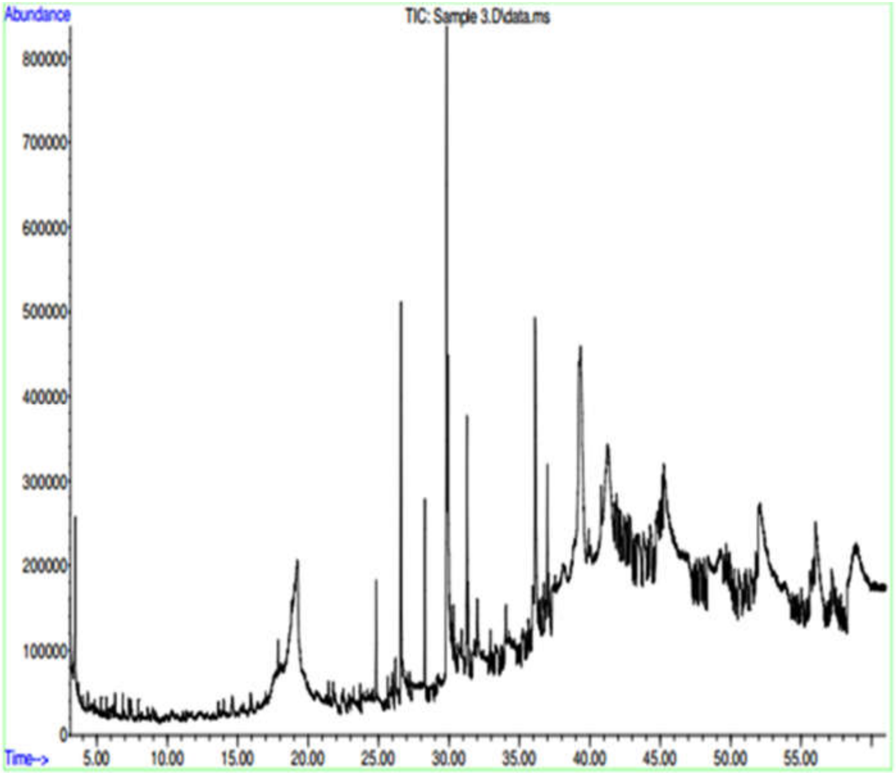

Figure S8: Biplot derived from multivariate statistical analysis

Biplot (axes F1 and F2: 60.41 %)

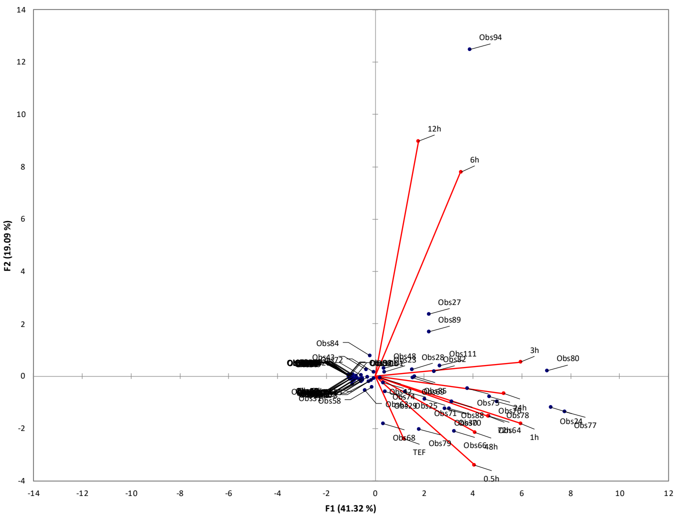

Table S1: Robustness parameters of method validation

**Parameters**		**GA-H Spiked to matrix (ng/mL)**	**% recovery (Mean ±%RSD)**
**Change in initial mobile phase composition**	Methanol:Water (5:95)	200	89.2±0.31
	Methanol:Water (10:90)	200	94.5±0.31
	Methanol:Water (15:85)	200	93.4±0.39
**Change in column temperature (°C)**	25	200	93.4±0.22
	30	200	94.3±0.31
	35	200	96.7±0.82
**Change in Flow Rate (mL/min)**	0.9	200	92.1±0.63
	1.0	200	93.4±0.43
	1.1	200	91.1±0.31

Table S2: Intra-day and inter-day precision data of method validation

**Metabolite**	**Conc. (ng/mL)**	**Inter-day**		**Intra-day**	
		**Mean Peak area ± SEM**	**%RSD**	**Mean Peak area ±SEM**	**%RSD**
**GA-H**	100	7036.45 ± 34.5	0.94	7089.15 ± 89.6	2.3
	200	13408.51± 245.5	2.67	13313.2 ± 132.5	1.98

Table S3. GC-MS profiling of TEF and unknown major compounds

**Compound**	**R_t (min)**	**Area**	**Area %**
Unknown	18.06	123512	0.01
Unknown	18.68	2572336	0.61
Unknown	18.83	6256562	1.49
Unknown	23.95	1320054	0.31
Unknown	24.59	1452603	0.34
Unknown	24.83	5279964	1.26
Unknown	25.66	1424588	0.34
Unknown	26.00	1607521	0.38
Unknown	26.21	2996981	0.71

Unknown	26.39	1217017	0.29
Unknown	27.05	1859504	0.44
Unknown	27.24	1409335	0.33
Unknown	27.41	495330	0.11
Unknown	29.32	258034	0.06
Unknown	34.27	2837571	0.68
Unknown	35.64	3471950	0.83
Unknown	35.95	7782425	1.86
Unknown	36.46	2992481	0.71
Unknown	36.73	5052431	1.21
Unknown	36.99	8990086	2.15
Unknown	37.41	6736045	1.61
Unknown	37.54	4161809	0.99
Unknown	38.12	4084632	0.97
Unknown	38.94	19518215	4.67
Unknown	39.24	35736288	8.56
Unknown	39.33	64132693	15.37
Unknown	40.92	7140276	1.71
Unknown	42.42	9407898	2.25
Unknown	42.51	5030878	1.20
Unknown	42.88	13167892	3.15
Unknown	43.16	3084226	0.73
Unknown	43.49	9158208	2.19
Unknown	44.63	3473825	0.83
Unknown	50.02	3056633	0.73
Unknown	51.98	5360617	1.28
Unknown	56.03	11588132	2.77
